# Comparison of cone beam computed tomography post-processing methods for online adaptive proton therapy of prostate cancer

**DOI:** 10.1016/j.phro.2025.100858

**Published:** 2025-11-01

**Authors:** Mislav Bobić, Daniel H. Bushe, Hoyeon Lee, Brian A. Winey, Jason A. Efstathiou, Harald Paganetti, Jennifer Pursley, Nils Peters, Lena Nenoff

**Affiliations:** a Department of Physics, ETH Zurich, Switzerland; b Department of Radiation Oncology, Massachusetts General Hospital and Harvard Medical School, Boston, MA, USA; c Department of Radiation Oncology, University of Washington & Fred Hutch Cancer Center, Seattle, WA, USA; d OncoRay – National Center for Radiation Research in Oncology, Faculty of Medicine, Dresden, Germany; e Helmholtz-Zentrum Dresden-Rossendorf, Institute of Radiooncology – OncoRay, Dresden, Germany; f Faculty of Medicine Carl Gustav Carus, TUD Dresden University of Technology, Dresden, Germany

**Keywords:** Proton therapy, CBCT, Proton dose calculation, Adaptive proton therapy, Prostate cancer

## Abstract

**Background and purpose:**

Although cone beam computed tomography (CBCT) enables online adaptive radiotherapy, its CT number accuracy may be insufficient for online adaptive proton therapy (OAPT). We compared proton dose distributions calculated directly on CBCT with calculations using additional CBCT image-processing methods in prostate cancer.

**Materials and methods:**

Retrospective proton plans were created for 10 prostate patients originally treated with 5-fraction online adaptive photon radiotherapy using CBCT. These plans were forward-calculated on each CBCT with four different approaches: (1) the clinical CBCT with a dedicated CT number calibration, (2) CBCT with histogram correction, (3) CT deformed to the CBCT, and (4) deformed CT with an air-cavity correction. Additionally, adaptive treatment using an OAPT workflow was simulated for each fraction and compared among the four CBCT-based methods. Dose-volume histograms (DVH) and related parameters were compared between the four methods for both non-adaptive and online adaptive treatment simulations.

**Results:**

Proton dose distributions were comparable across all CBCT-based strategies, with median differences of up to 1% for all DVH metrics compared to a reference method. Larger differences were observed for doses calculated directly on the CBCT for patient geometries deviating from the CBCT-specific calibration. Despite these differences, all four methods indicated the dosimetric benefits of OAPT over non-adaptive treatment.

**Conclusion:**

An advanced CBCT system enables proton dose calculations performed directly on the CBCT, demonstrating sufficient accuracy for integration into an OAPT workflow. Additional CBCT-based post-processing techniques are recommended to maximize the dosimetric benefit of plan adaptation in all patient populations.

## Highlights

• A cone beam computed tomography system was calibrated for proton therapy.
• Image post-processing methods were evaluated for online adaptive dose calculation.
• Median differences across methods were within 1% for key dose-volume metrics.
• All methods indicated dosimetric benefits of plan adaptation in prostate cases.

## Graphical abstract

**Figure:**
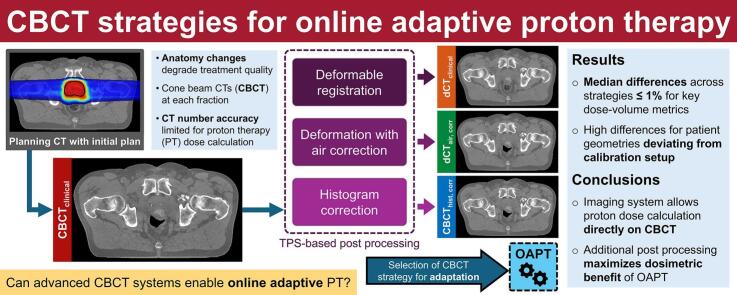

## 1 Introduction

Daily online treatment plan adaptation has become clinically available for photon radiation therapy (RT) with the integration of streamlined software solutions combining linear accelerators with magnetic resonance imaging (MRI) [1,2] or high-quality cone beam computed tomography (CBCT), leading to improved dose conformity during treatment [[3], [4], [5], [6]]. Online adaptive proton therapy (OAPT) is starting to be clinically adopted, but currently relies on in-room CT imaging [7,8]. The spatial and financial constraints associated with in-room CT imaging have hampered the widespread clinical adoption of OAPT. While CBCT solutions can be mounted to the proton gantry, on a robotic C-arm, or directly to the couch [9], the resulting images suffer from artifacts, making them currently unsuitable for direct proton dose calculation [10,11]. Although various promising CBCT correction methods for proton dose calculation have been investigated [12], none have been approved by the US Food and Drug Administration (FDA) or the EU Medical Device Regulation (MDR) for proton therapy, preventing clinical application.

In adaptive photon RT, clinical workflows use commercial systems that permit dose calculation and online plan adaptation directly on CBCT images [[13], [14], [15]]. Other solutions have been applied by correcting CT numbers or by transferring the planning CT to the daily anatomy. One approach for CT number correction is to match the CBCT intensity histogram to the histogram of a reference CT in an iterative process [16]. Alternatively, the reference CT is deformed to the CBCT with or without corrections for low-density regions (lung, air) [17,18]. Although these CBCT-based post-processing methods enable online adaptive photon RT, deformation-induced uncertainties are difficult to quantify and can diminish the benefits of plan adaptation [19]. Furthermore, the applicability of these methods to OAPT remains uncertain due to the inherent sensitivity of proton plans to stopping-power mapping errors and the resulting range uncertainty.

In this retrospective study, we investigate the potential use of a high-end CBCT imaging system for proton dose calculations and OAPT of prostate cancer patients. We evaluate the applicability of these images for proton dose calculations by comparing them to three different CBCT-based post-processing methods available in treatment planning systems (TPS). We analyze the need for plan adaptation and evaluate its potential dosimetric benefits based on the daily anatomy. The utilization of clinically available systems is an important step towards the future implementation of CBCT-based OAPT.

## 2 Methods and materials

### 2.1 Patient data and treatment planning

Ten patients diagnosed with malignant neoplasm of the prostate were included in this retrospective study, Institutional Review Board (IRB) number 2016P002036 (Table 1). All patients had received curative treatment with online adaptive stereotactic body RT (SBRT) using Varian’s Ethos machine (Varian Medical Systems, Palo Alto, CA, USA) between May 2023 and December 2023 at the Massachusetts General Hospital (MGH). The prescribed dose to the clinical target volume (CTV, comprising the prostate gland and seminal vesicles) was 37.5 Gy, delivered in 5 fractions over two weeks (7.5 Gy per fraction). For each fraction, a high-quality CBCT was acquired using the HyperSight imaging solution (field-of-view 538 mm, scan length 300 mm) [13], enabling direct dose calculation on the CBCT for online adaptive RT. Target and organ-at-risk (OAR) structures were automatically generated with the Ethos software during each session and manually corrected by clinical staff as needed.

**Table 1:** Overview of the patent cohort originally treated with 37.5 Gy in 5 fractions with online adaptive SBRT on the Ethos machine.

Patient #	Age group	BMI/kg m^−2^	CTV size/cm^3^	Staging
1	60–65	23.4	61.5	Stage I
2	70–75	30.6	48.2	Stage IIB
3	70–75	27.8	67.2	Stage IIB
4	65–70	26.4	48.5	Stage IIB
5	65–70	28.6	45.6	Stage I
6	65–70	24.8	51.9	Stage IIB
7	70–75	51.5	57.1	Stage IIC
8	60–65	24.0	40.8	Stage IIIA
9	60–65	26.7	46.1	Stage I
10	60–65	28.0	35.0	Stage IIB

Intensity-modulated proton therapy (IMPT) treatment plans were created retrospectively in RayStation 2023B (v14.0.100.0, RaySearch Laboratories, Stockholm, Sweden) using the same CTV prescription as for the initial photon treatments (37.5 Gy in five fractions), assuming a constant relative biological effectiveness (RBE^3^) of 1.1. Plans were calculated and optimized on the original planning CTs (pCT). These images were acquired with a GE Discovery CT590 wide-bore scanner (General Electric Medical Systems, Milwaukee, WI), with a voxel size of 0.98 × 0.98 × 1.25 mm^3^. Each plan consisted of two laterally opposed beams (90° and 270°). The planning target volume (PTV) was defined as in the initial photon treatment: 3-mm expansion anteriorly and posteriorly from the CTV, and 5-mm expansion in all other directions. Nominal plans were optimized using multi-criteria optimization to meet the clinical goals (Table S1). Planned dose distributions were calculated and optimized with the Monte Carlo engine in RayStation (IonMonteCarlo v5.4) on a 2.0 × 2.0 × 2.0 mm^3^ dose grid. Each treatment plan was approved by a board-certified medical physicist, and a radiation oncologist verified the planning approach.

### 2.2 Image calibration and post-processing

**Fig. 1:**
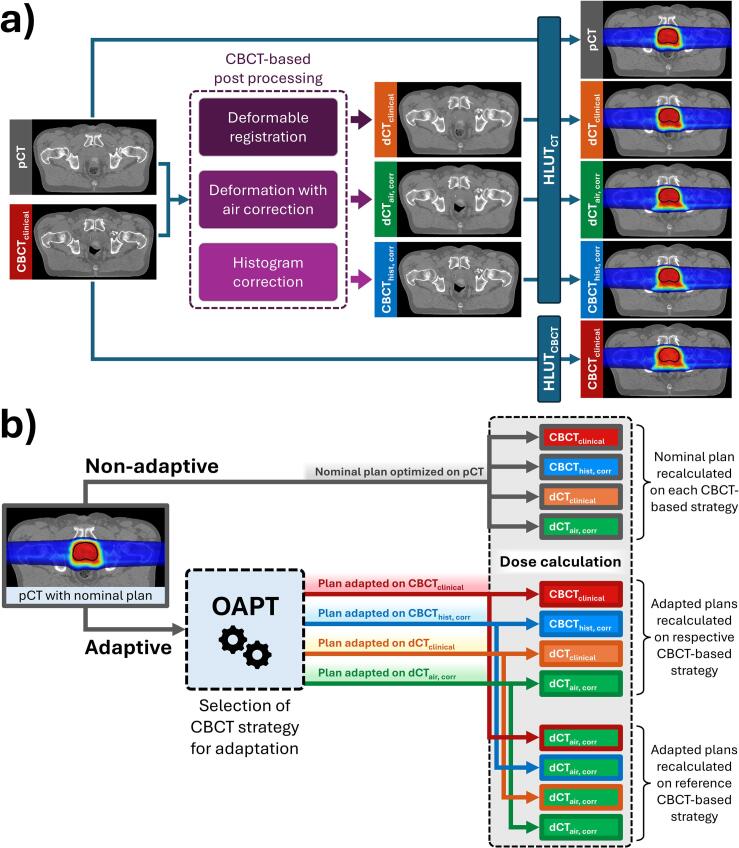
Study design schematic. **a)** CBCT-based post-processing strategies resulting in four different images, each using one of the two HLUTs for dose calculation. **b)** Simulation of treatment delivery, showing all evaluated dose calculations. Colored arrows indicate distinct treatment plans, while colored boxes show which CBCT-based fraction image is used for dose calculation.

To obtain proton range information from the daily CBCT, four different post-processing approaches were followed (Fig. 1a): (1) a direct application of a CBCT-scanner-specific translation Hounsfield look-up table (HLUT) from CT number to stopping power ratio (SPR) of the CBCT (HLUT_CBCT_), (2) a modification of the CBCT histogram to match the pCT, and two deformable image registration approaches to match the pCT, without (3) and with (4) air bubble correction, respectively. For methods 2–4, a HLUT_CT_ calibration was obtained for the planning CT scanner. The same calibration method and setup as for the CBCT was used to avoid bias.

1) HLUT calibration on HyperSight system: CBCT_clinical_

The large-field HyperSight system contains an enlarged X-ray panel (86 × 43 cm^2^), allowing full in-plane coverage of the patient and a focused grid, substantially reducing the dependence of CT numbers on patient geometry. Together with an advanced scatter correction in post-processing [20], the resulting image impressions are similar to those of fan-beam CT [21]. All CBCTs were acquired with a tube voltage of 140 kVp, a tube current of 125 mA, and an exposure time of 5.8 s using the ‘Acuros’ reconstruction. The voxel size ranged from 0.88 × 0.88 × 2.0 mm^3^ to 1.05 × 1.05 × 2.0 mm^3^, with a corresponding CTDI_vol_ ranging from 18.78 mGy to 19.87 mGy, depending on the patient geometry. For the calibration of HLUT_CBCT_, a Gammex Tissue Characterization Phantom 467 (Sun Nuclear, Middleton, WI, USA) was scanned. To ensure scatter balance in the z-direction, the phantom was immersed in a water tank approximating the phantom geometry. The HLUT_CBCT_ was then defined following the EPTN guide on HLUT calibration [22].

2) Histogram-correction approach: CBCT_hist, corr_

CT numbers were corrected using an iterative method implemented in RayStation, aiming to match the histogram of the CT numbers in the CBCT to that of the CT numbers in the pCT [18]. First, a CT number correlation between CBCT and pCT is established via a linear fitting procedure in a joint histogram. A difference map is created to mask image regions where CT numbers deviate, resulting in a correction map by iteratively adjusting the low-frequency signal. This method has been clinically validated for photon dose calculation [23].

3) Deformable image registration on the scanner: dCT_clinical_

In ETHOS’ CBCT scanner software, an elastic deformation model is used to match the pCT to the CBCT [17], generating the deformed dCT_clinical_.

4) Deformable image registration with air bubble correction: dCT_air, corr_

The deformation from pCT to CBCT in dCT_air, corr_ is different from the implementation in dCT_clinical_ and includes two steps: First, an image-intensity-based deformable image registration is applied (ANACONDA in RayStation) [24]. Then, to account for anatomical changes due to air cavities, a correction is applied in low-density regions (ρ < 0.6 g/cm) with substantial density changes (Δρ > 0.3 g/cm) between the two images [18]. CT numbers in the deformed pCT are replaced with those from CBCT_hist corr_.

### 2.3 Dose calculation and treatment simulation

We evaluated proton dose distributions calculated on different CBCT-based fraction images. Dose calculations on CBCT_clinical_ used the dedicated HLUT_CBCT_ calibration, while all others used HLUT_CT_ (Fig. 1a). All dose distributions were calculated with the recently validated GPU-based Monte Carlo code MOQUI [[25], [26], [27]], using the exact same beam model as for nominal treatment plan optimization with a statistical uncertainty of 2 % for the PTV dose. Treatment simulation considered both non-adaptive and online adaptive delivery, realized with the following two workflows (Fig. 1b):

1) Non-adaptive delivery

The nominal treatment plan was forward-calculated on the CBCT-based image after aligning its position to the plan isocenter with a 3-degree-of-freedom (3-DOF) registration between pCT and CBCT_clinical_ (Plastimatch [28]). The resulting registration was visually verified and used to shift each of the four images for a given fraction. Non-adaptive delivery was simulated for each of the four CBCT-based fraction images.

2) Online-adaptive delivery

Using the same 3-DOF registration as for non-adaptive delivery, we applied an OAPT workflow previously developed at MGH [29]. This online dose restoration workflow employs MOQUI to calculate the dose-influence matrix, followed by beamlet weight reoptimization to restore the originally planned dose distribution deformed to the daily image. For this study, we extended our workflow from head-and-neck patients [[30], [31], [32], [33], [34], [35]] to the pelvic region by allowing reoptimization of all beamlets rather than just a subset. Each adapted plan was calculated on its corresponding CBCT-based fraction image. To minimize bias and validate the consistency of the adapted plans, each plan was additionally forward-calculated on dCT_air, corr_, designated as the reference method with the most accurate representation of the daily anatomy based on our initial observations.

### 2.4 Evaluation metrics

Evaluation was based on dose-volume histograms (DVH) and the following clinically evaluated parameters: D_98%_ and $D_{1cm^{3}}$ for the CTV, (voxel) maximum dose to the bladder, D_50%_ and $D_{3cm^{3}}$ for the rectum. Each fraction dose was scaled by a factor of 5 (i.e., total number of fractions) to allow direct comparison with the planned dose. Gamma pass rates (3 %/3 mm) were evaluated for all fractions, with an analysis threshold of 3.75 Gy (RBE), i.e., 10 % of the prescription.

## 3 Results

### 3.1 Impact of CBCT-based strategy on dose calculation

**Fig. 2:**
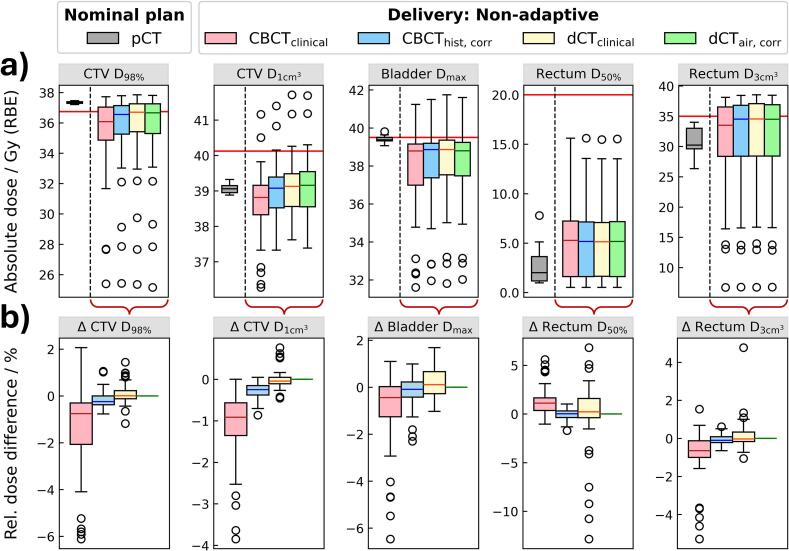
Nominal plans forward-calculated on all CBCT-based fraction images (i.e., non-adaptive delivery) for the different strategies. **a)** Absolute dose-volume metrics comparing dose delivery across different fraction images (n = 50), with the nominal metrics from plans optimized on the pCT (n = 10) shown on the left of each subplot. **b)** Fraction-wise relative differences between dose-volume metrics of each CBCT-based strategy and a reference method (dCT_air, corr_), indicating larger discrepancies for CBCT_clinical_ compared to other methods. The boxplots show the median and the 25th and 75th percentiles (lower and upper hinges). Whiskers extend to the smallest and largest values within 1.5 times the interquartile range from the hinges; points beyond are shown as outliers.

Dose-volume metrics for the non-adaptive delivery (Fig. 1b, non-adaptive route) are summarized in Fig. 2: Compared to the nominal plan, target coverage decreased while rectum dose increased in most cases, indicating a degradation of plan quality due to anatomical changes. These trends were consistent across all four CBCT-based strategies, with CBCT_clinical_ showing the largest deviations on average. This is further exemplified in the fraction-wise dose metric differences against the reference method dCT_air, corr_ (Fig. 2b). While large outliers (up to 6.1 % for the CTV and up to 12.8 % for OARs) were observed for some fractions, the median dose differences remained within 1 % across all dose-volume metrics, indicating comparable dose calculation in most cases. The corresponding 3 %/3 mm gamma pass rates confirmed these results, averaging 98.6 %, 100.0 %, and 99.9 % for CBCT_clinical_, CBCT_hist, corr_, and dCT_clinical_, respectively (Table S2).

**Fig. 3:**
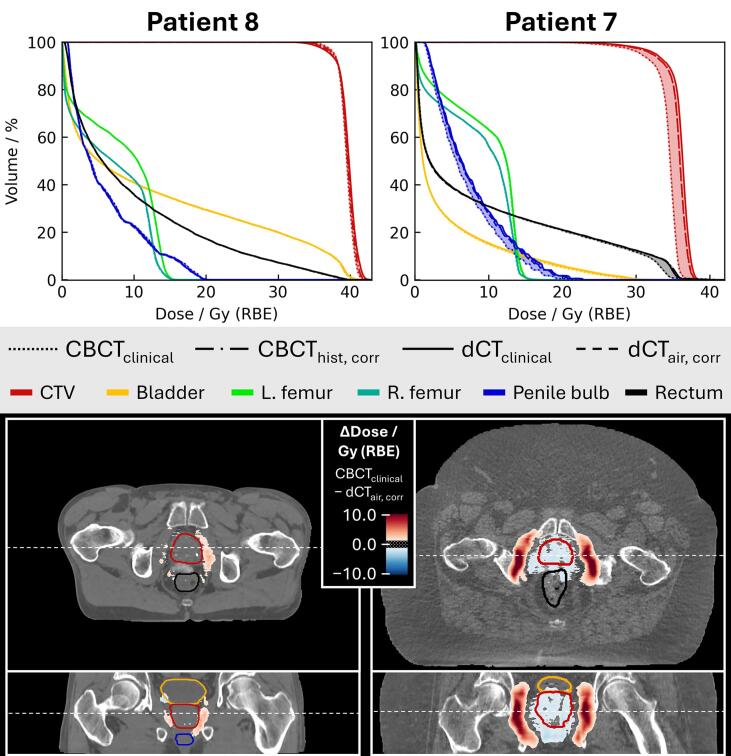
Differences in proton dose calculation for two patients with different BMI. **Top:** Fraction doses (scaled by a factor of 5) compared in terms of DVHs for each CBCT-based image of a single fraction. **Bottom:** Corresponding dose differences (CBCT_clinical_ minus dCT_air, corr_) superimposed on the axial and coronal views of CBCT_clinical_. Differences between −1 and 1 Gy (RBE) are not visualized. The dashed lines indicate the locations of the respective axial and coronal slices. Overshooting of the dose calculated on CBCT_clinical_ is evident, resulting in a considerably lower predicted target coverage for patient 7 compared to dCT_air, corr_.

Comparison of two example patients (Fig. 3) showed very similar DVHs across all structures for patient 8 in the normal-weight range, whereas for the obese patient 7, large discrepancies were observed in the CTV-DVH calculated on CBCT_clinical_ compared to the other strategies. The respective voxel-wise dose differences (Fig. 3, bottom), indicate an overshoot of the dose on CBCT_clinical_ compared to the dose calculated on the reference method (dCT_air, corr_). For patient 8, voxel-wise differences did not exceed 3 Gy (RBE), whereas for patient 7, differences of up to 15 Gy (RBE) were observed surrounding the CTV at the proton beam’s end-of-range. Additionally, for patient 7, the overshooting on CBCT_clinical_ resulted in a considerably lower dose in the CTV compared to the reference, also visible in the DVHs.

### 3.2 Impact of CBCT-based strategy on plan adaptation

**Fig. 4:**
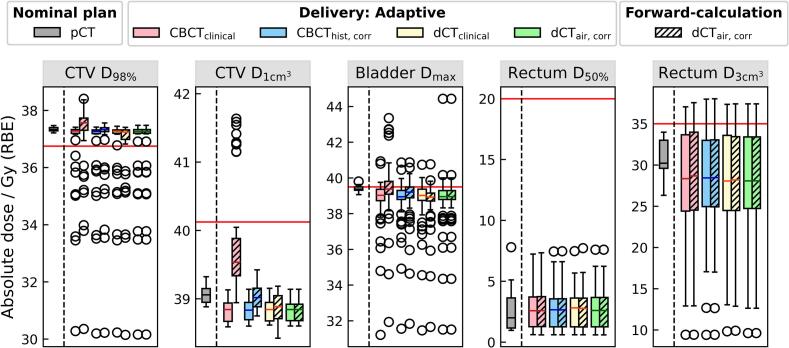
Dose-volume metrics evaluated for online adaptive delivery (n = 50) across all CBCT-based fraction images, compared to the nominally planned doses (n = 10). Plain boxplots represent the final dose calculation of the online adapted plans on their respective fraction images (i.e., calculation of the adapted plan on the same image on which it was optimized). Striped boxplots show additional forward-calculations of the adapted plans on a reference method (dCT_air, corr_). By definition, both the plain and striped boxplots for dCT_air, corr_ contain the exact same data.

Dose-volume metrics of the OAPT workflow (Fig. 1b, adaptive route) are summarized in Fig. 4, comparing adaptive dose delivery with the nominally planned dose. For each CBCT-based strategy, DVH metrics were calculated by simulating the adapted plan on its corresponding image (plain boxplots), resulting in similar values across the different strategies. These values were consistent with the nominal plan in terms of target coverage (except for outliers), but showed a higher D_50%_ and lower $D_{3cm^{3}}$ for the rectum. The additional forward-calculations on the reference method dCT_air, corr_ (striped boxplots) showed comparable values across all CBCT-based strategies for OARs. However, for the CTV, higher doses were observed for plans adapted on CBCT_clinical_ compared to the other methods, with a notably increased $D_{1cm^{3}}$ and slightly increased D_98%_.

## 4 Discussion

We present the first evaluation of proton dose calculations performed directly on CBCTs approved for online adaptive RT, compared with three TPS-based CBCT post-processing strategies. Direct CBCT-based dose calculation was comparable to these strategies, supporting its future suitability for OAPT on a high-end imaging system. While deviations occurred for patient geometries substantially different (i.e., larger) from the calibration setup, all approaches consistently indicated a dosimetric benefit of OAPT over non-adaptive delivery.

All investigated approaches showed a similar plan dose degradation when not considering the daily anatomy in treatment planning (i.e., non-adaptive delivery), with CBCT_clinical_ showing the largest variations across dose-volume metrics (Fig. 2). The CBCT-based strategies comprised a trade-off between CT number accuracy and the anatomy of the day: The two deformation approaches preserve the high CT number accuracy as indicated by the dose comparisons, but only partially represent the daily anatomy [3]. Conversely, the histogram-corrected CBCT accurately represents the patient’s anatomy, but only mimics the CT numbers of the planning CT. The small shift toward CTV underdose in CBCT_clinical_ compared to CBCT_hist, corr_ indicates a slight overestimation of the tissue’s CT numbers. Uncertainties introduced by deformation are intrinsically difficult to quantify compared to imaging-related uncertainties [19,24]. The performance of CBCT_clinical_ directly reflects the anatomy of the day, but depends on CT number stability across patients and fractions. Image quality was sufficient for consistent proton dose calculation, though larger patients may require dedicated calibration, consistent with Kunnen *et al.* [15]. While previous studies have shown the feasibility of CBCT-based methods using in-house scatter-correction algorithms or synthetic CT generation to enable OAPT [11,12,[36], [37], [38], [39], [40], [41]], our study focused on TPS-based CBCT post-processing methods, as this facilitates widespread clinical application.

CT numbers in conventional fan-beam CT scans are dependent on the effective diameter of the patient, which in clinical practice needs to be considered either with an additional beam hardening correction and/or with body-size-specific calibrations [24]. In CBCT, this effect is amplified, resulting in highly geometry-dependent CT numbers [42,43]. CBCT_clinical_ was calibrated specifically for beam hardening conditions mimicking those of a pelvis, but the scans do not incorporate advanced beam hardening correction for bone tissue. For cases deviating from the calibration geometry, this directly resulted in a calculated proton beam overshoot and corresponding CTV underdose (Fig. 3). In conventional CT imaging, the most common approach is to use a single HLUT covering all scenarios [44]. To meet this standard, advanced image correction methods in CBCT imaging are needed. Furthermore, we did not observe an impact of imaging noise on the performance of the four methods, which is in alignment with several studies showing that CT noise (within realistic ranges) has a negligible impact on proton dose calculation accuracy [33,45,46].

Simulating OAPT demonstrated the feasibility of our beamlet weight-tuning approach for prostate patients (Fig. 4). However, individual fractions exhibited insufficient CTV coverage even after adaptation. This directly correlated with the patient’s anatomy of the day: Optimization objectives for rectum sparing were highly weighted and, due to its close proximity to the CTV, resulted in reduced target coverage in these fractions. This is most evident for patient 8, fraction 4, with a CTV D_98%_ ≈ 30  Gy (RBE) across all four strategies (Fig. S3), highlighting the prioritization of rectum sparing over target coverage. For consistency, we did not vary planning objectives on a per-fraction basis, which is possible in clinical practice.

Additional forward-calculations of the different adapted plans on dCT_air, corr_ validated the consistency of adaptive delivery across all methods (Fig. 4, striped boxplots) except for plans optimized on CBCT_clinical_. These plans resulted in CTV overdose for some fractions, a direct consequence of the overshoot and associated CTV underdose predicted in non-adaptive delivery (Fig. 3). To compensate for the (predicted) insufficient target coverage, the OAPT optimization increased the fluence of beamlets hitting the CTV. These cases highlight the risk of adapting plans on a suboptimal image; for example, the obese patient 7 showed a higher CTV $D_{1cm^{3}}$ for plans optimized on CBCT_clinical_ (compare Fig. 2a with Fig. 4).

High-quality, 3D imaging in the treatment position to fully account for anatomical changes and daily patient setup is a key missing link for online adaptive proton therapy [47]. The worldwide first OAPT implementation [7] and the first clinical trial on OAPT [8] used in-room CTs. However, most proton clinics lack the space for in-room CT imaging, making CBCT-based OAPT desirable [48]. This would also allow repeat imaging directly in the treatment position without additional movement from the CT scanner to the treatment couch.

Spectral CT imaging, such as dual-energy CT or photon counting CT, is gaining traction due to improved soft-tissue contrast and tissue quantification capabilities [49,50] and could provide additional information for beam hardening correction [51,52]. The general feasibility of spectral CBCT has been demonstrated in experimental or small-bore setups [[53], [54], [55]]. Implementing such a correction could overcome the current dependence of CT numbers on patient size.

The similar performance of the deformable and direct CBCT-based approaches shows that even basic correction approaches provide a relevant dosimetric benefit for plan adaptation. If substantial anatomical changes close to the CTV are expected (e.g., rectal cavity filling), deformation approaches specifically considering air cavities are required. For patients matching the calibration phantom geometry, direct dose calculation and adaptation on the CBCT proved to be beneficial over not adapting at all. Remaining uncertainties could be addressed by increasing range robustness settings (or similarly extended margins) during online adaptation [56].

In this work, we focused on the dosimetric impact of patient geometry and correction methods for proton dose calculation as the relevant endpoint. Since the CBCT scanner setup was not optimized for application in proton therapy, it may contain additional uncertainties such as daily CT number variations that do not affect photon dose calculation. As a proof-of-concept, we used a CBCT system that provides the best image quality in an FDA-approved setup. However, the system is mounted to the linac gantry. For proton therapy, a gantry-mounted CBCT would have a much greater distance to the isocenter, resulting in more scatter artifacts [37,41]. In an open system, the rotation speed and reproducibility would be reduced. Similarly, a couch-mounted solution is conceivable, but would have a much slower image acquisition, introducing motion artifacts. Upright proton treatment may open new possibilities for a CBCT gantry [57,58].

In conclusion, we have shown that proton dose calculations performed directly on a high-quality CBCT are feasible for OAPT, while additional CBCT-based post-processing is recommended to cover all patient scenarios and maximize the dosimetric benefit of plan adaptation.

## Funding statement

This work was funded by the National Cancer Institute (NCI), Grant Number R01 CA229178. The article processing charge was funded by ETH Zurich.

## CRediT authorship contribution statement

**Mislav Bobić:** Conceptualization, Methodology, Software, Validation, Formal analysis, Investigation, Data curation, Writing – original draft, Writing – review & editing, Visualization. **Daniel H. Bushe:** Methodology, Investigation, Data curation, Writing – review & editing. **Hoyeon Lee:** Methodology, Software, Investigation, Writing – review & editing. **Brian A. Winey:** Writing – review & editing, Supervision. **Jason A. Efstathiou:** Writing – review & editing. **Harald Paganetti:** Writing – review & editing, Supervision, Funding acquisition. **Jennifer Pursley:** Writing – review & editing, Supervision. **Nils Peters:** Conceptualization, Methodology, Validation, Formal analysis, Investigation, Data curation, Writing – original draft, Writing – review & editing. **Lena Nenoff:** Conceptualization, Methodology, Validation, Formal analysis, Investigation, Data curation, Writing – original draft, Writing – review & editing, Project administration, Funding acquisition.

## Declaration of competing interest

The author is an Editorial Board Member/Editor-in-Chief/Associate Editor/Guest Editor for this journal and was not involved in the editorial review or the decision to publish this article. Dr. Nils Peters is an Editorial Board Member of this journal. All other authors have no conflict of interest to declare. This work was funded by the National Cancer Institute (NCI), Grant Number R01 CA229178. The article processing charge was funded by ETH Zurich.

## Footnotes

3 Gy (RBE) denotes the RBE-weighted absorbed dose as defined in ICRU Report 93.
