# Supplementary material for: Comparison of cone beam computed tomography post-processing methods for online adaptive proton therapy of prostate cancer
Source: Phys Imaging Radiat Oncol. 2025 Nov 1;36:100858. doi: 10.1016/j.phro.2025.100858 (PMC12663649; doi:10.1016/j.phro.2025.100858)
Supplement: Supplementary Data 1 [file mmc1.pdf]

Table S1: Clinical goals defined during plan optimization. For PTV-based goals, % refers to the prescribed dose. D_{max} refers to the maximum dose to a single voxel, whereas all other D-values refer to the minimum dose to the most irradiated sub-volume of a structure.

**Structure**	**Clinical Goal**
CTV	$D_{98\%} \geq 98\%$
PTV	$D_{98\%} \geq 100\%$
	$D_{99\%} \geq 95\%$
	$D_{max} \leq 107\%$
Bladder	$D_{max} \leq 39.5$ Gy (RBE)
	$D_{50\%} \leq 19$ Gy (RBE)
Rectum	$D_{max} \leq 39.5$ Gy (RBE)
	$D_{3cm^3} \leq 35$ Gy (RBE)
	$D_{10\%} \leq 33$ Gy (RBE)
	$D_{20\%} \leq 30$ Gy (RBE)
	$D_{50\%} \leq 20$ Gy (RBE)
Penile bulb	$D_{max} \leq 37.5$ Gy (RBE)
	$D_{50\%} \leq 21$ Gy (RBE)
Left femur	$D_{10cm^3} \leq 16$ Gy (RBE)
	$D_{1cm^3} \leq 21$ Gy (RBE)
Right femur	$D_{10cm^3} \leq 16$ Gy (RBE)
	$D_{1cm^3} \leq 21$ Gy (RBE)
Urethra	$D_{max} \leq 39.5$ Gy (RBE)

Table S2: Gamma pass rates (3%/3 mm) comparing doses calculated using the reference method $dCT_{air, corr}$ with doses calculated using the other three CBCT-based strategies.

Gamma pass rate / %		$CBCT_{clinical}$	$CBCT_{hist, corr}$	$dCT_{clinical}$
**Mean (min–max)**		**98.6 (90.5–100.0)**	**100.0 (100.0–100.0)**	**99.9 (98.9–100.0)**
Patient 1	Fraction 1	99.86	100.00	99.66
	Fraction 2	99.93	100.00	99.76
	Fraction 3	99.92	100.00	99.85
	Fraction 4	99.89	100.00	99.70
	Fraction 5	99.84	100.00	99.60
Patient 2	Fraction 1	99.85	100.00	99.71
	Fraction 2	99.90	100.00	99.97
	Fraction 3	99.87	99.99	99.99
	Fraction 4	99.96	100.00	99.87
	Fraction 5	99.98	100.00	99.99
Patient 3	Fraction 1	100.00	100.00	99.54
	Fraction 2	99.99	100.00	100.00
	Fraction 3	100.00	100.00	100.00
	Fraction 4	100.00	100.00	100.00
	Fraction 5	99.99	100.00	100.00
Patient 4	Fraction 1	96.68	100.00	98.92
	Fraction 2	97.21	100.00	100.00
	Fraction 3	97.25	100.00	100.00
	Fraction 4	96.61	100.00	100.00
	Fraction 5	96.52	100.00	100.00
Patient 5	Fraction 1	99.46	100.00	100.00
	Fraction 2	98.41	100.00	100.00
	Fraction 3	99.54	100.00	100.00
	Fraction 4	99.49	100.00	100.00
	Fraction 5	98.50	99.99	100.00
Patient 6	Fraction 1	99.66	99.99	100.00
	Fraction 2	99.87	99.99	100.00
	Fraction 3	99.82	99.99	100.00
	Fraction 4	99.71	99.98	100.00
	Fraction 5	99.72	99.99	100.00
Patient 7	Fraction 1	91.49	100.00	99.58
	Fraction 2	92.63	100.00	99.91
	Fraction 3	91.75	100.00	99.97
	Fraction 4	91.55	100.00	99.96
	Fraction 5	90.50	100.00	99.95
Patient 8	Fraction 1	99.98	100.00	100.00
	Fraction 2	99.99	100.00	99.94
	Fraction 3	100.00	100.00	100.00
	Fraction 4	99.99	100.00	99.99
	Fraction 5	100.00	100.00	99.62
Patient 9	Fraction 1	99.97	100.00	99.68
	Fraction 2	99.80	100.00	99.99
	Fraction 3	99.96	100.00	100.00
	Fraction 4	99.97	100.00	99.59
	Fraction 5	99.75	100.00	100.00
Patient 10	Fraction 1	99.06	100.00	100.00
	Fraction 2	99.60	100.00	100.00
	Fraction 3	99.67	100.00	100.00
	Fraction 4	99.27	100.00	100.00
	Fraction 5	99.81	100.00	100.00

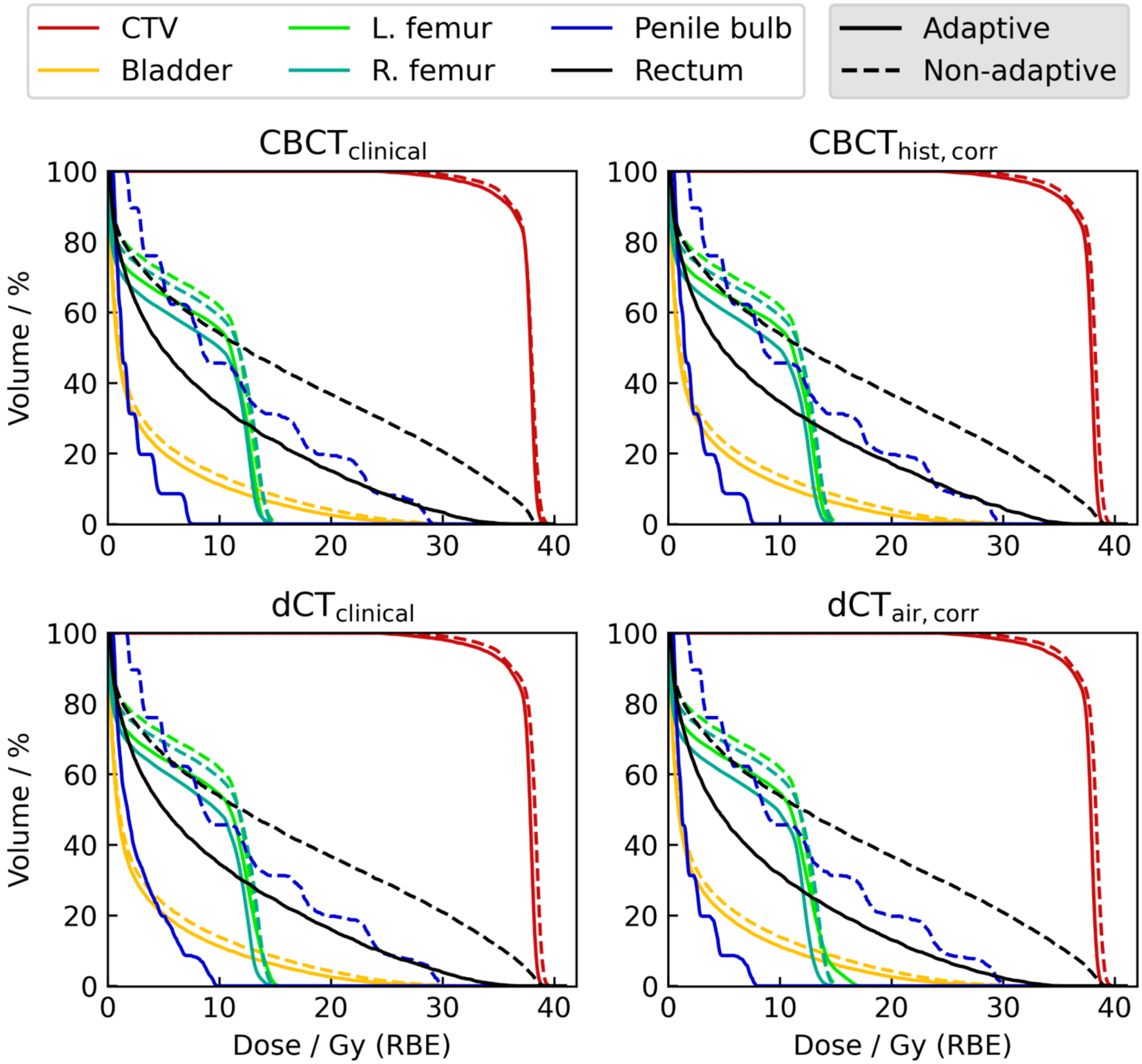

Figure S3: DVHs for both adaptive and non-adaptive delivery, highlighting the prioritization of rectum sparing over CTV coverage for this fraction (patient 8, fraction 4) in OAPT optimization. These trends are consistent across all four CBCT-based strategies.
